# Keratin Scaffold Formulation Impacts rhBMP-2 Biodistribution and Bone Regeneration in a Rat Femur Defect Model

**DOI:** 10.1007/s13770-025-00747-4

**Published:** 2025-08-04

**Authors:** Justin M. Saul, Judy S. Bohnert, Molly O’Brien, Saif Alnuaimi, Troy P. Carnwath, Quinn Dunivan, Douglas W. Coffin, Thomas L. Smith

**Affiliations:** 1 Department of Chemical, Paper and Biomedical Engineering, Miami University, 650 East High Street, Oxford, OH 45056 USA; 2 Department of Orthopaedic Surgery, Medical Center Boulevard, Wake Forest University School of Medicine, Winston-Salem, NC 27157 USA; 3 Present Address: Department of Biomedical Engineering, College of Engineering and Applied Science, University of Cincinnati Bioscience Center, Cincinnati, OH USA; 4 Present Address: Cincinnati Children’s Hospital, Cincinnati, OH USA; 5 Present Address: Tawam Hospital, Al Ain, Abu Dhabi, UAE; 6 Present Address: Department of Emergency Medicine, Carolinas Medical Center, Charlotte, NC USA

**Keywords:** Biomaterials, Natural materials, Fracture, Nonunion, Recombinant human bone morphogenetic protein

## Abstract

**Background:**

Treatment for nonunion in long bones remains a clinical need. Collagen sponges loaded with recombinant human bone morphogenetic protein 2 (rhBMP-2) are potential grafts but have limited FDA-approved applications due to safety concerns associated with rapid collagen resorption and burst release of rhBMP-2 *in vivo*. This work investigates keratin proteins obtained from human hair as a potential rhBMP-2 biomaterial carrier. Keratins are an appealing carrier because the extent of disulfide crosslinking can be modulated by the form of keratin present, thus allowing control over the rate of scaffold degradation.

**Methods:**

The two forms of keratin used to formulate carriers were reductively extracted keratin called kerateine (KTN) that can form disulfide crosslinks and oxidatively extracted keratin called keratose (KOS) that does not form disulfide crosslinks. Five formulations of freeze-dried keratin scaffolds containing variable amounts of KOS and KTN were fabricated and implanted into a critically-sized rat femur defect model.

**Results:**

A 50:50 KOS:KTN formulation with rhBMP-2 showed the same level of bone bridging, bone mineral density, and bone volume as collagen with rhBMP-2 by 8 weeks as determined by μ-CT. Scaffolds with the 50:50 KOS:KTN or 100% KTN showed approximately fourfold higher retention of fluorescently-labeled rhBMP-2 at the implant site 1, 3, or 7 days post-implant compared to collagen or 100% KOS scaffolds. The increased retention with 50:50 KOS:KTN or 100% KTN correlated with decreased levels of fluorescent rhBMP-2 in distal organs.

**Conclusions:**

Keratin scaffolds could provide comparable levels of bone regeneration as collagen carriers with improved safety profiles suitable for use in long bone nonunions.

**Supplementary Information:**

The online version contains supplementary material available at 10.1007/s13770-025-00747-4.

## Introduction

The rate of nonunion in bone fractures is approximately 2% [1], but 20% rates of nonunion have been reported in diaphyseal fractures of long bones [2, 3]. Donor site morbidity from cortical or trabecular autografts and less-than-satisfactory outcomes from allogeneic demineralized bone matrix have driven the search for alternative systems that can promote safe and effective bone regeneration [4, 5]. Non-cellular or “off-the-shelf” approaches to promote bone healing may be particularly valuable from both clinical and regulatory perspectives.

Recombinant human bone morphogenetic protein 2 (rhBMP-2) delivery from collagen sponges in the form of Medtronic’s Infuse Bone Graft is an off-the-shelf product that has been shown to promote robust bone regeneration. Collagen-mediated delivery of rhBMP-2 showed promising results in open tibial fracture [6], which led to FDA-approval via the humanitarian exemption mechanism. rhBMP-2 delivery from collagen sponges has also been used “off-label” for other long bones including femur and humerus [7]. While off-label use is permitted, more widespread use of these products in long bone nonunions has been limited, in part due to safety concerns including edema, inflammation, ectopic bone formation, and bone cysts [8].

Burst release of the rhBMP-2 underlies safety concerns, but properties of collagen such as rapid degradation *in vivo* likely play a key role as well. Materials that can better regulate rhBMP-2 delivery while still achieving fracture healing would be beneficial. Natural polymer carriers may be advantageous if they contain cell binding motifs that promote osteoconductivity. [9–12] One drawback that has been associated with natural materials is the lack of tunable degradation that can be achieved with synthetic polymers or the need for chemical modification of the natural polymers to achieve tunable degradation [13].

Keratins are a class of natural polymers that can be obtained from various sources including human hair. These materials that may provide some of the benefits of both natural (osteoconductivity) and synthetic (tunable rates of degradation) polymers. A salient feature of keratins is the high prevalence of cysteine residues in their primary structure, which allows the inherent formation of disulfide crosslinks. Reductive extraction of keratin yields a form referred to as kerateine (KTN), which leaves the thiol group of cysteine intact and available for disulfide crosslinking. Oxidative extraction gives a form referred to as keratose (KOS), but this extraction method yields a sulfonic acid group on the cysteine residues that prevents KOS from forming disulfide crosslinks. It has previously been demonstrated that modification of the number of cysteine residues available for disulfide crosslinking can be achieved by simply mixing the KOS and KTN forms of keratin in different ratios [14, 15]. By varying the ratios of KOS and KTN in keratin materials, the rates of degradation of materials composed of keratin can be controlled in a manner reminiscent of synthetic polymers. It has also been shown that the rates of keratin degradation achieved by manipulation of the levels of disulfide crosslinking impact the amount and quality of bone in a heterotopic mouse model [15].

The hypothesis guiding the current study was that healing and safety could be improved by keratin carriers with tunable rates of degradation relative to collagen carriers for rhBMP-2 in a long bone model of nonunion. This model consists of a critically-sized defect in the left femur with internal fixation. We investigated bridging, bone volume and bone mineral density by micro-computed tomography (μ-CT) at 4 and 8-week time points and mechanical properties at the 8-week time point. We also investigated the retention of rhBMP-2 at the defect site by using a near-IR fluorescent label on the rhBMP-2 to determine if keratin carriers could mitigate rhBMP-2 burst release as a potential way to improve safety profiles.

## Materials and methods

### Materials

Recombinant human bone morphogenetic protein 2 (rhBMP-2), sterile water, and collagen sponges were obtained from Medtronic (Minneapolis, MN) as part of an XX Small Infuse Synthetic Bone Graft kit. Keratin in both the keratose (KOS) and kerateine (KTN) forms was obtained from KeraNetics, LLC and used without further modification. DyLight800 (DL800), AlexaFluor488 NHS ester, and Nalgene 890 FEP tubing were obtained from ThermoFisher Scientific.

### Keratin scaffold preparation

Keratin scaffolds were prepared by first formulating 15% w/v keratin hydrogels with varying ratios of KOS and KTN by methods similar to those previously described [14, 15]. Powders of keratose, kerateine or mixtures of keratose and kerateine were weighed in sterile fashion into 50 mL conical tubes. Then, 500 μL of sterile water with or without rhBMP-2 was added to the keratin powders and then thoroughly mixed with a sterile spatula followed by vortexing to ensure even loading of the rhBMP-2 within the keratin. Each of the keratin formulations was prepared in the same manner. In some experiments, the rhBMP-2 was labeled with AlexaFluor 488 (AF488) or DL800 (see “Fluorescent Labeling of rhBMP-2” section, below). For *in vitro* studies, 100 μL of the keratin hydrogels were used instead of scaffolds due to absorption of water into the scaffolds that impacted the accuracy of initial time points. For *in vivo* studies, the keratin hydrogels were packed into 1.5 cm long × 4.8 mm inside diameter FEP tubes with a small amount of air pressure applied by syringes attached to each end to ensure complete packing of keratin in the tubes. The keratins were then allowed to gel overnight. The resulting gels were frozen at − 80 °C for at least 24 h and then lyophilized on a Labconco Freezone lyophilizer (Labconco, Kansas City, MO). We refer to these freeze-dried keratins as keratin scaffolds. After lyophilization, the scaffolds for *in vitro* or *in vivo* studies were stored in sterile containers under vacuum until use. Typical preparations for the keratin scaffolds are shown in Table 1. For the *in vitro* studies, the starting amount of rhBMP-2 was 10 μg (in 100 μL volume of keratin). As described below, the actual amount of rhBMP-2 implanted at the femur defect site for the *in vivo* studies was 14.2 μg.

**Table 1:** Typical preparation of keratin hydrogels with different KOS and KTN masses

Formulation	Mass of KOS (mg)	Mass of KTN (mg)	Total mass of keratin (mg)	Volume of water or 100 μg/mL rhBMP-2 (with or without DL 800 or AF488) (μL)
KOS	75	0	75	500
70:30 KOS:KTN	52.5	22.5	75	500
50:50 KOS:KTN	37.5	37.5	75	500
30:70 KOS:KTN	22.5	52.5	75	500
KTN	0	75	75	500

### Rat femur defect model and implantation of biomaterials

Femur defect surgeries were conducted with the approval of the Miami University Institutional Animal Care and Use Committee (IACUC). Approximately 3-month old Sprague–Dawley male rats (average weight of 371.5 g) were randomly assigned to experimental groups. Rats were induced with isoflurane (3–5%) and then maintained with isoflurane via nosecone at 1–3%. The left hindlimb was shaved over the femur. The left femur was exposed by making incisions through the skin and then the muscle along the longitudinal axis of the femur. A custom-built internal fixator device was designed and implanted as described previously [16, 17]. In brief, a pilot hole was drilled into the left femur with a wire. The fixator device was placed midway between the proximal and distal ends of the femur. Two gold-plated stainless steel (grade 303) screws were used to secure the fixator to the bone. An 8 mm segment of bone was removed by using a pneumatic reciprocating saw. The bone tissue was removed from the defect site and the defect site was rinsed with sterile saline to remove any bone fragments. The size of the defect was made with guides in the fixator set at 8 mm.

Collagen or keratin biomaterials were then implanted at the site of the defect, with the exception that negative controls received no biomaterial implants (empty). Keratin scaffolds were prepared as described above. The freeze-dried scaffolds were removed from the FEP tubes and the scaffolds were then cut to length in the surgical field at a length slightly longer than 8 mm. We assumed the the keratin with rhBMP-2 was well-mixed. Based on the volume of the keratin scaffolds implanted (142 mm^3^ or 0.142cm^3^) and the rhBMP-2 concentration used to prepare the hydrogels prior to freeze-drying (100 μg/mL), the implanted mass of rhBMP-2 was approximately 14.2 µg.

For collagen, an 8 mm segment of collagen was cut by scissors from the XX Small Infuse kit. 14.2 μg of rhBMP-2 in water (142 μL of 100 μg/mL rhBMP-2) was then added to ensure that the mass of rhBMP-2 was the same for collagen as for keratin. The rhBMP-2 was allowed to adsorb to the collagen for 15 min according to the manufacturer’s recommendation. The swollen collagen sponge was then implanted at the defect site.

No additional means were used to secure collagen sponges or keratin scaffolds at the implant site. The site was then closed in layers (muscle followed by skin) and the skin was stapled. Sutures and staples were removed within 14 days following surgery.

In the retention study described below, the rhBMP-2 was labeled with DL800 before being added to keratin or collagen biomaterials. All other methods remained as described above.

### μ-CT image collection

For μ-CT imaging, animals from Miami University were transported to the University of Cincinnati Vontz Imaging Center at 4 and 8 weeks after implantation as approved on the Miami University IACUC protocol. Upon arrival at the Vontz Center, rats were temporarily placed on an imaging protocol approved by the University of Cincinnati IACUC. Rats were anesthetized with isoflurane (3–5% to induce, then maintained at 1—1.5% via nosecone). Animals were placed in the prone position with their left hindlimb extended. Rats were imaged on a Siemens μ-CT unit with a single rotation about the axis with 80 kV voltage, 500 microAmp current, 450 ms exposure at 360 steps to achieve voxel resolution of ~ 52 cubic microns. After imaging, rats were transferred back to the Miami University protocol and returned to Miami University for housing (4-week time point) or humane euthanasia (8-week time point).

### μ-computed tomography image analysis of bone regeneration

DICOM images from the μ-CT scans were reconstructed in Osirix or Horos DICOM-viewers with 3D volume rendering. Bridging of the defects was determined by visualization of the resulting scans. Regions of interest (ROI) were drawn manually in Osirix in the defect region from every tenth image and interpolated to calculate the volume of new bone growth. Bone mineral density (BMD) was determined by gray scale standards run with each μ-CT scan and determined by gray-scale analysis in ImageJ (NIH, USA). Each of these processes were conducted by a blinded investigator.

### Bone harvest and mechanical testing

After humane euthanasia, left and right (uninjured contralateral) femurs from all rats except one were immediately dissected and measured by caliper at hip, knee, and central diaphysis. In total, 5 left and right femurs were used for mechanical testing (N = 5) with the other femur (N = 1) used for histology (see below). Mechanical testing was conducted by the four-point bend method in which the superior side of the bone was placed in the downward direction. The bottom fixtures were set at L = 26 mm apart and top fixtures at (L-2a) = 10 mm apart. The hip was placed to the right side. Specimens were measured on an Instron Mechanical tester apparatus equipped with a 1000 N load cell. The bones were then pre-loaded in compression to 0.5N from the top fixture. The bones were compressed at a crosshead speed of 1 mm/min until fracture. Load and displacement were measured and converted to engineering stress and strain measurements as described previously [18].

In brief, $\sigma =\frac{FLc}{4I} \text{and } \varepsilon =\frac{6cd}{a(3L-4a)}$where σ sigma is engineering stress, ε is engineering strain, F is the measured force in compression, L is the length between the bottom fixtures, c is the maximum distance from the cross-sectional center of mass (taken to be the minor axis of the bone) to the outside edge of the bone, d is the displacement of the top supports relative to the bottom supports, a is the distance from one top fixture to one bottom fixture (8 mm), and I is the second moment of the cross-sectional area relative to the horizontal axis. Modulus was determined as the maximum slope of the stress vs. strain curves. A running average was used to obtain the maximum slope.

Due to ectopic growth in some animals, we assumed that the diameter of the specimen at the sites of load application was the average of the knee and hip widths and diameters in an oval shape. Further, we could not reliably measure the inner and outer bone diameters and therefore assumed a solid cylinder for modulus calculations. This assumption as well as the use of a four-point bend likely underestimates the true modulus [18], but we report the modulus as the percentage of the contralateral limb in order to normalize the data.

### Histology

One bone sample was used for histological imaging. The tissue was fixed for three days in 10% Neutral Buffered Formalin (NBF) and then decalcified for three days in Immunocal (Decal Chemical Corporation, Tallman, NY). Decalcification was stopped with Cal-Arrest (Decal Chemical Corporation, Tallman, NY). Bone tissue was soaked in 30% sucrose overnight, and then snap frozen in OCT Compound for frozen sectioning. Tissue was sectioned in the longitudinal direction on a Leica cryostat. Sections were collected on positively-charged slides, dried overnight, stained with Masson’s Trichrome (Master Tech, Lodi, CA) or hematoxylin and eosin (H&E), and imaged at the Miami University Center for Advanced Microscopy and Imaging (CAMI) on a Nikon 300 microscope equipped with a 2X objective. Images of individual sections were stitched together into mosaics by using GNU Image Manipulation Program (GIMP).

### Fluorescent labeling of rhBMP-2

1.35 mL of 1 mg/mL of rhBMP-2 was prepared in DI water. 37.8 μL of DyLight800 (DL800) NHS ester in DMF was added and the solution was mixed in the dark by magnetic stirring at room temperature for 1 h. Two (2) μL of 0.5 M HCl was then added to stop the reaction. The solution was then transferred to a 3500 Da MWCO Slide-A-Lyzer membrane (ThermoFisher Scientific) and dialyzed exhaustively with three changes of DI water. The DL800-labeled rhBMP-2 was removed from the dialysis cassette, measured at 280 nm for determining rhBMP-2 concentration against a standard curve. The resulting DL800-rhBMP-2 was then diluted to 1 mg/mL in water. The DL800 concentration was determined by absorbance readings at 777 nm. A mole dye:mole protein ratio of 2 was typical (i.e., average of 2 dye molecules per rhBMP-2 molecule). This solution of DL800-rhBMP-2 was stored at − 20 °C until use. The DL800-rhBMP-2 was added (“spiked”) to unlabeled rhBMP-2 at 1 part in 15 (1:15) ratio and then used for preparation of collagen sponges or keratin scaffolds for implantation into the femur defect model, as described above.

For an *in vitro* study, the same method was used to label rhBMP-2 with the exception that AF488 NHS ester was used as the fluorophore.

### *In vitro* release of AF488-labeled rhBMP-2

Keratin hydrogels were prepared as described in the “Keratin Scaffold Preparation” section where 100 μg/mL AF488-labeled rhBMP-2 was used and 100 μL the keratins hydrogels were placed into 1.5 mL tubes. 150 μL of PBS was placed on top of each keratin hydrogel formulation and removed at specified time points (1.5 h, 3 h, 6 h, 12 h, 24 h and then daily through 7 days). Keratin hydrogels that did not contain AF488-labeled rhBMP-2 were used as negative controls. Fluorescence was detected at excitation/emission wavelengths of 485 nm/528 nm. Autofluorescence from keratin proteins was determined from the negative controls (no rhBMP-2) and this amount of signal was subtracted from samples with AF488-labeled rhBMP-2. The concentration was determined by comparison to a standard curve of known concentrations of AF488-labeled rhBMP-2.

#### Femur retention and biodistribution of rhBMP-2 after implantation

To determine the amount of DL800-rhBMP-2 retained at the defect site, we first developed a standard curve for known amounts of DL800-rhBMP-2 at the defect site. 15% (w/v) KOS scaffolds were used for creating the standard curve for DL800 fluorescence. The DL800-rhBMP-2 was prepared at rhBMP-2 concentrations of 100, 50, 25, 12.5, 6.25, 3.125, 1.5625 or 0 μg/mL, which correspond to 14.2, 7.25, 3.625, 1.813, 0.906, 0.453, 0.227, and 0 μg implanted mass of rhBMP-2. The scaffolds were implanted as described in the femur defect model studies, above. Rats were randomly assigned to one of the DL800-rhBMP-2 concentrations for the standard curve and 3 rats (N = 3) were imaged for each DL800-rhBMP-2 concentration. After closure of the implant site, rats were humanely euthanized and frozen until transport to the University of Cincinnati Vontz Imaging center. The incision site was opened to expose the defect site and implanted materials. The rats were placed on a Bruker *in vivo* Multispectral Imaging System. One X-ray and one optical image were taken to confirm location of the femur defect and then the rats were imaged in the near-IR range by using a 730/790 Ex/Em filter set. Images were collected and pixel intensity was integrated with the Magnetic Lasso Tool in Photoshop to correlate DL800-rhBMP-2 concentration to fluorescence intensity. A standard curve was then created relating DL800-rhBMP-2 concentration with the measured pixel intensity, where it was assumed that the DL800-rhBMP-2 concentration was the same as initially implanted.

To determine the retention (where it was assumed that non-retained rhBMP-2 had been released from the implant site) of DL800-rhBMP-2 from keratin or collagen carriers at the implant site, rats were implanted with KOS, KTN, 50:50 KOS:KTN, or collagen containing 14.2 μg DL800-rhBMP-2. Rats were randomly assigned to each treatment group and four rats (N = 4 at Days 1 and 3, N = 5 at Day 7) were implanted for each formulation at each time point. Preparation of collagen and keratin as well as implantation in the femur defect model as described above. Rats were humanely euthanized and transported to the University of Cincinnati Vontz Imaging Center at 1, 3, or 7 days after surgical implantation of the scaffolds with rhBMP-2 (spiked at 1:15 with DL800-rhBMP-2). The incision site was opened to expose the defect site and imaged as described above for the standard curve. Pixel intensity was determined in Photoshop and the DL800-rhBMP-2 concentration was determined from the standard curve generated above. After imaging the femur defect site, the brain, liver, spleen, kidneys, heart and lungs were removed and imaged on the Bruker *in vivo* multispectral imaging system.

#### Statistical methods

Animal studies were randomized. Imaging and data analysis were conducted by blinded observers. We confirmed previous studies showing that keratin scaffolds lacking rhBMP-2 do not lead to bridging in this critically-sized defected model. To reduce the number of animals in the study, we combined results from empty (no treatment), keratose only (no rhBMP-2) and kerateine only (no rhBMP-2) and treated these as near replicates for statistical comparisons. These are shown as “Combined Controls” in the figures and for statistical comparisons, but where appropriate we have also shown the results for the Combined Control groups. Comparisons between groups were conducted with analysis of variance followed by Tukey’s honestly significant different post-hoc test.

## Results

### Bone regeneration at 4 and 8 weeks

**Fig. 1:**
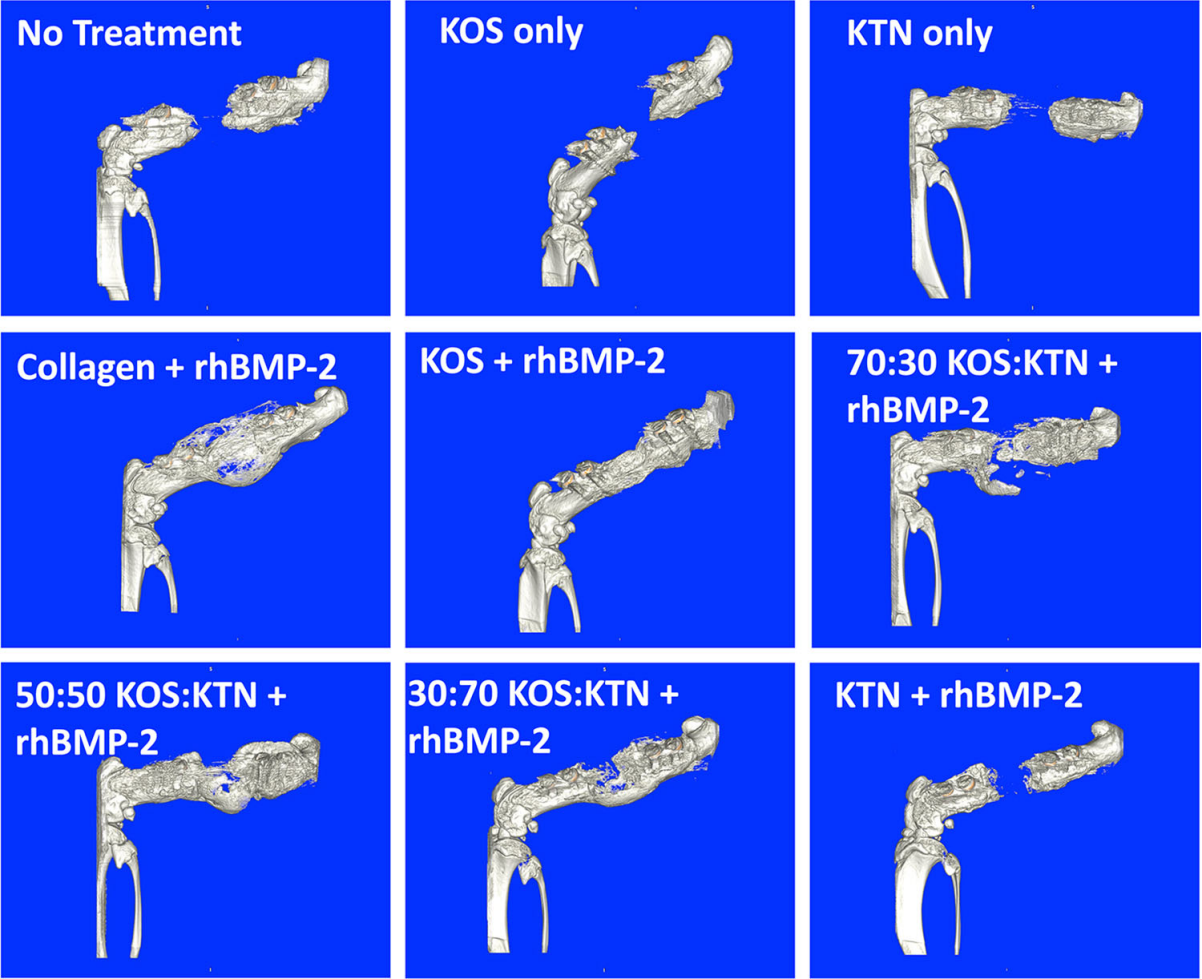
Lateral view μ-CT renderings of left femurs 4 weeks after implantation (or no treatment/empty control)

**Fig. 2:**
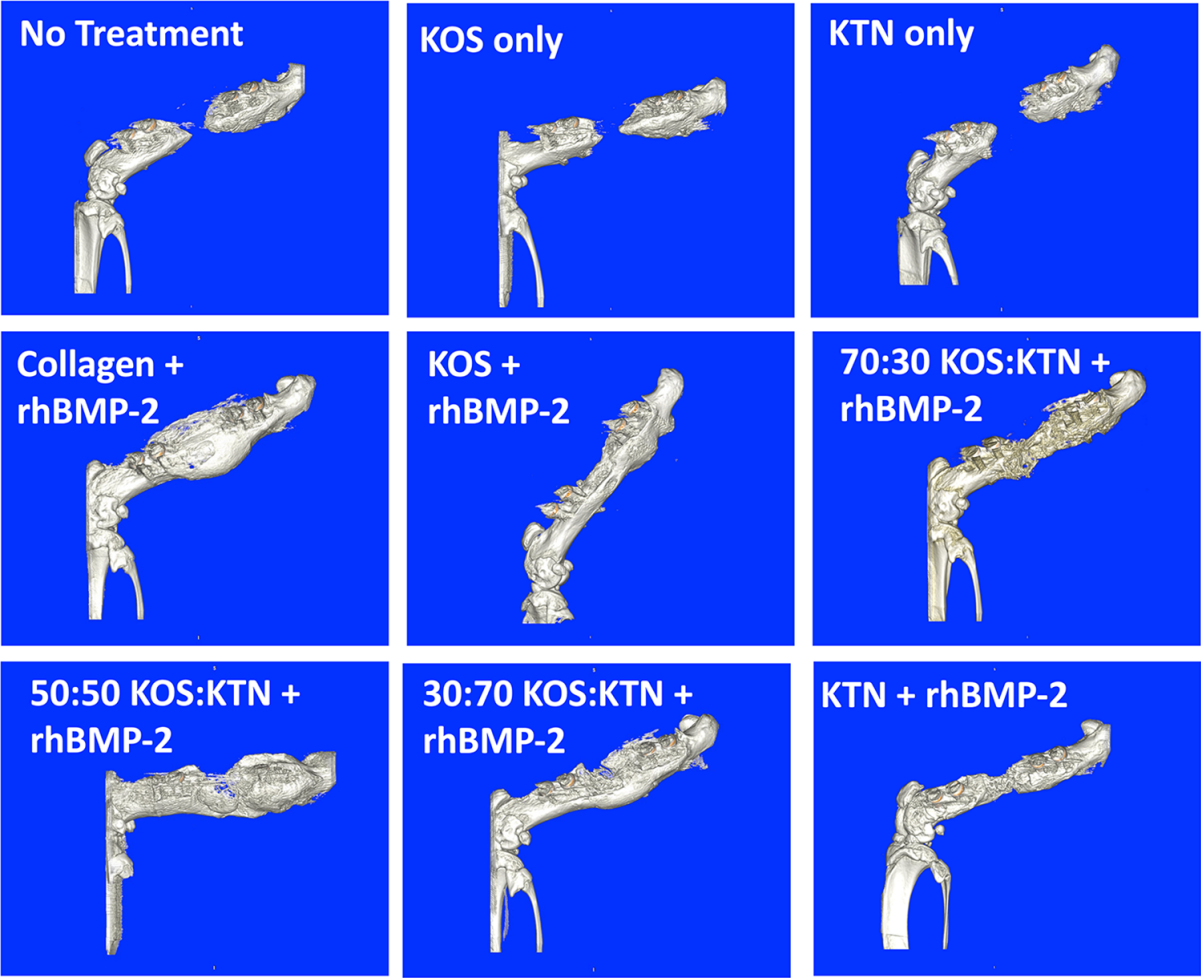
Lateral view μ-CT renderings of left femurs 8 weeks after implantation (or no treatment/empty control)

**Fig. 3:**
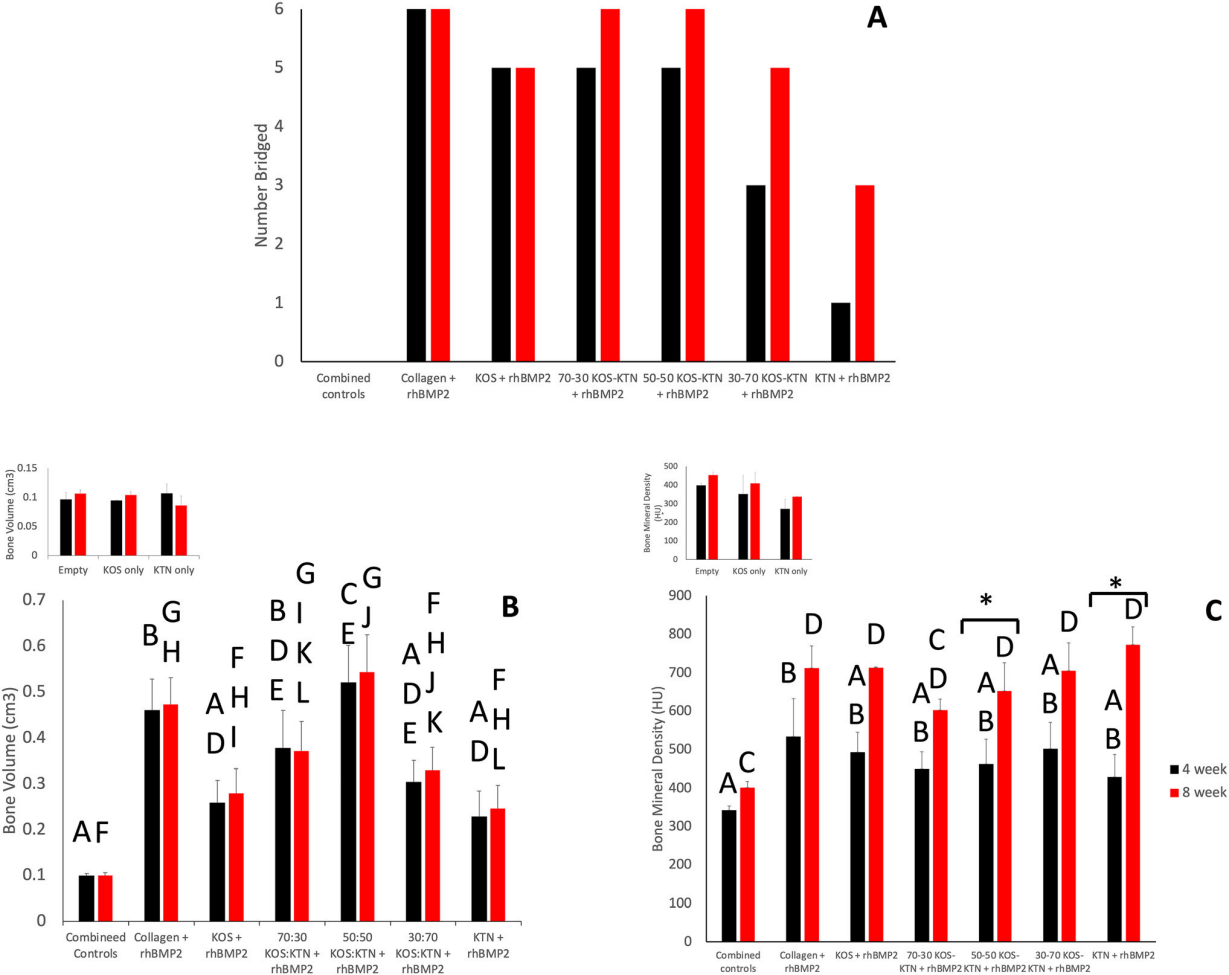
Analysis of μ-CT renderings of left femurs at 4 and 8 weeks after implantation. **A** Number of rats that showed bone bridging for each treatment group. **B** Bone volume for each treatment group. **C** Bone mineral density for each treatment group. N = 2 for empty, N = 3 for KOS-only, N = 2 for KTN-only, and these groups are combined as near replicates shown as Combined Controls. The results for these groups (empty, KOS-only, KTN-only) individually are shown as inserts for (B) and (C). None of the Combined Controls showed bridging. N = 6 for all other treatment groups. Error bars are standard error of the mean. Statistical analyses were conducted for the 4-week and 8-week time points for (B) and (C). Groups that share a letter were not found to be statistically different (*P* > 0.05) while groups that do not share a letter were found to be statistically different (*P* < 0.05). There were also no differences within groups between the 4 and 8 week time points except for BMD for the 50:50 and KTN groups, as indicated by * (*P* < 0.05)

Figures 1 and 2 show lateral views of μ-CT 3D renderings for one representative animal from each treatment group at 4 weeks and 8 weeks. Figure 3 shows analysis for animals in each treatment group at both 4 and 8 weeks, respectively.

Untreated rats (empty defect) showed that the defect was still present at both 4 weeks (Fig. 1) and 8 weeks (Fig. 2). Although bird feather keratin montmorillonite scaffolds have been reported to support osteogenic differentiation of marrow-derived stem cells and promote healing in a rat calvarial defect model [19], we observed that KOS-only (no rhBMP-2) and KTN-only (no rhBMP-2) treatments still had large defects at both 4 and 8 weeks (Figs. 1 and 2, respectively), consistent with prior studies with KOS[16] and KTN[20] derived from human hair showing minimal bone formation in the absence of rhBMP-2. Empty, KOS-only, and KTN-only did not show bridging in any animals by 8 weeks (Fig. 3A). These results confirm the critically-sized nature of this model of nonunion. Lack of bone formation in the absence of rhBMP-2 was the reason that we treated empty, KOS-only, and KTN-only as near replicates (Combined Controls) for statistical purposes, thereby reducing the number of animals required for the study.

Collagen with rhBMP-2 showed bridging by 4 weeks in all animals, but the bone had a large callous with a thin cortex (Fig. 1). The bone was less porous but still showed a larger callous area by 8 weeks (Fig. 2). Bridging was not as rapid for keratin formulations as for collagen. Collagen + rhBMP-2 showed bridging in all 6 animals by 4 weeks but none of the keratin formulations with rhBMP-2 had bridging in all animals by the 4-week time point (Fig. 3A). However, by 8 weeks both the 70:30 KOS:KTN and 50:50 KOS:KTN formulations with rhBMP-2 showed bridging in 6/6 animals. The 30:70 KOS:KTN and KTN formulations with rhBMP-2 had two more animals with bridged defects by 8 weeks than 4 weeks with 30:70 + rhBMP-2 going from 3 to 5 bridged and the KTN + rhBMP-2 going from 1 to 3 bridged from 4 to 8 weeks. Only the KOS + rhBMP-2 did not show additional animals with bone bridging at 8 weeks (bridging in 5/6) compared to the 4-week time point (5/6).

### Bone mineral density and bone volume at 4 and 8 weeks

The empty, KOS-only, and KTN-only (i.e., no rhBMP-2) formulations showed minimal bone volume in the defect site at 4 or 8 weeks, further confirming the critically-sized nature of this model. The collagen + rhBMP-2, 70:30 KOS:KTN + rhBMP-2, and the 50:50 KOS:KTN + rhBMP-2 groups reached statistical significance (P < 0.05) for bone volume compared to the Combined Controls at both the 4 and 8-week timepoints. Surprisingly, the KOS, KTN, and 30:70 KOS:KTN formulations with rhBMP-2 did not reach the level of statistical significance compared to the Combined Controls. Further, the 50:50 KOS:KTN + rhBMP-2 had statistically greater bone volume than the KOS + rhBMP-2 and KTN + rhBMP-2 formulations. (Fig. 3B). This is likely because formulations with more KOS or KTN with rhBMP-2 showed lower levels of callous outside of the defect site (Figs. 1 and 2), similar to previous observations with KTN in a mandible model [20]. None of the other keratin formulations with rhBMP-2 had bone volumes as large as collagen at 4-week or 8-week time points. It is noteworthy that the KOS + rhBMP-2 and KTN + rhBMP-2 formulations showed lower bone volumes than the keratin mixtures with rhBMP-2 (70:30, 50:50, and 30:70 KOS:KTN) at both time points.

At the 4-week time point, only the collagen with rhBMP-2 had significantly greater BMD than the Combined Controls (empty, KOS without rhBMP-2, and KTN without rhBMP-2). However, by the 8-week time point, the collagen and all keratin formulations with rhBMP-2 except the 70:30 KOS:KTN formulation had significantly greater BMD values than the Combined Controls (Fig. 3C). There were no statistical differences between any of the formulations containing rhBMP-2 (collagen, KOS, 70:30 KOS:KTN, 50:50 KOS:KTN, 30:70 KOS:KTN, or KTN) at the 4-week or 8-week time points. We note that BMD values increased from the 4-week to the 8-week time points for all groups, but only the increases in the 50:50 KOS:KTN + rhBMP-2 and KTN + rhBMP-2 groups were statistically significant. In addition, the increases were larger in those animals treated with rhBMP-2 compared to the Combined Controls.

### Bone mechanical properties at 8-week time point

One sample for each treatment group was not used for mechanical testing so that it could be used for histology. One sample from the 70:30 KOS:KTN + rhBMP-2 was inadvertently broken when lowering the top fixture, so this group had N = 4. The remaining groups had N = 5 for mechanical testing. None of the empty control, KOS-only or KTN-only samples could be mechanically tested due to a lack of connectivity. Therefore, the max load and moduli were taken as 0 for these groups.

**Fig. 4:**
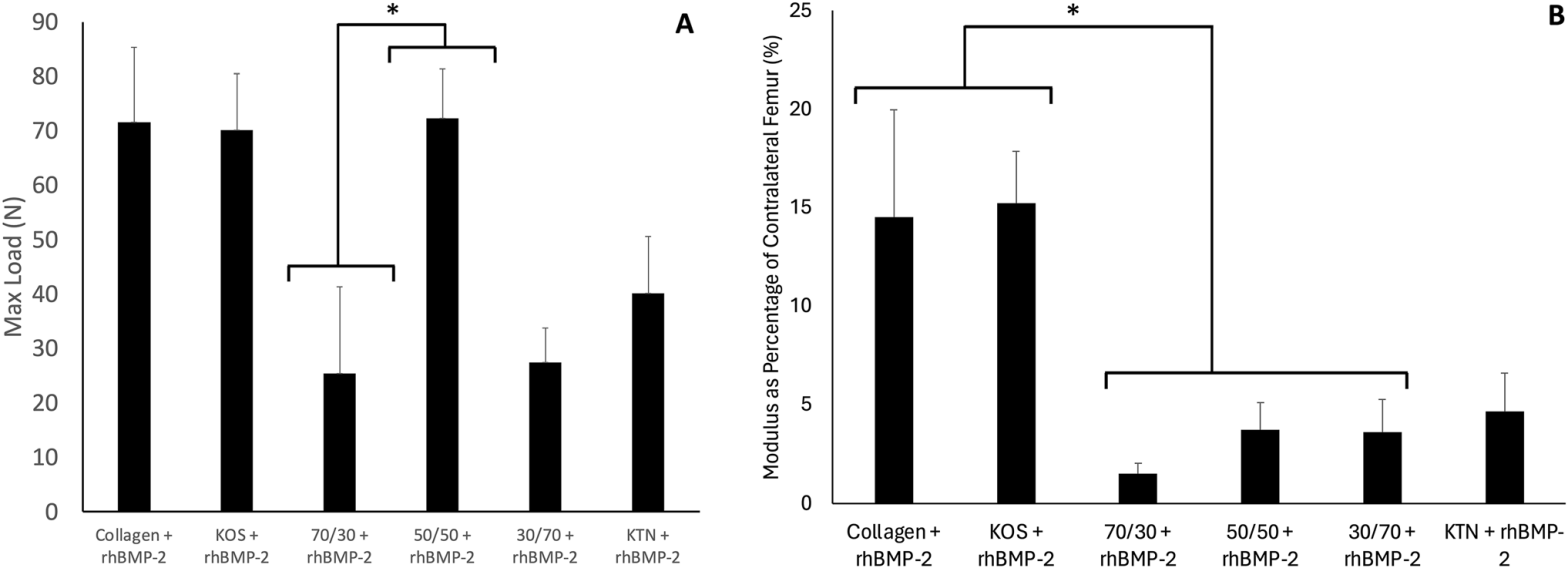
Mechanical properties of left femurs at time of explantation (8 weeks after implantation) as determined by 1 mm/min with 1000 N load cell. **A** Max load at fracture. **B** Elastic modulus as determined from engineering stress and engineering strain. N = 5 all groups except 70:30 KOS:KTN where the sample broke in the Instron prior to testing. None of the groups had all 6 femurs from experimental animals subjected to mechanical testing because one sample was taken for histology. Max load and modulus could not be determined for Combined Controls because the tissue at the defect site was too weak to load into the mechanical testing apparatus. Error bars denote standard error of the mean

The 30:70 KOS:KTN + rhBMP-2 had a statistically significantly lower max load than the 50:50 KOS:KTN + rhBMP-2 load, but none of the other comparisons reached the level of statistical significance due to variability in the max loads. As shown in Fig. 4A, the KOS + rhBMP-2 and 50:50 KOS:KTN + rhBMP-2 had max loads of ~ 70 N, which was close to that for collagen + rhBMP-2. The 70:30 KOS:KTN + rhBMP-2 and 30:70 KOS:KTN + rhBMP-2 had max loads less than half of the collagen, KOS, and 50:50 KOS:KTN formulations with rhBMP-2 while the KTN + rhBMP-2 was 40% less than the collagen, KOS, and 50:50 KOS:KTN formulations with rhBMP-2. However, none of these differences reached statistical significance.

The collagen + rhBMP-2 and KOS + rhBMP-2 groups had statistically greater elastic moduli than each of the other keratin formulations with rhBMP-2 except for the KTN + rhBMP-2 (Fig. 4B). However, the collagen + rhBMP-2 group was not statistically different than the KOS + rhBMP-2 group. There was considerable intra-group variability in the elastic moduli but we observed that the moduli values largely reflected the μ-CT observations. That is, animals with weak or no bridging as observed by μ-CT tended to show lower modulus values.

### In vitro rhBMP-2 release and *in vivo* rhBMP-2 retention and biodistribution

**Fig. 5:**
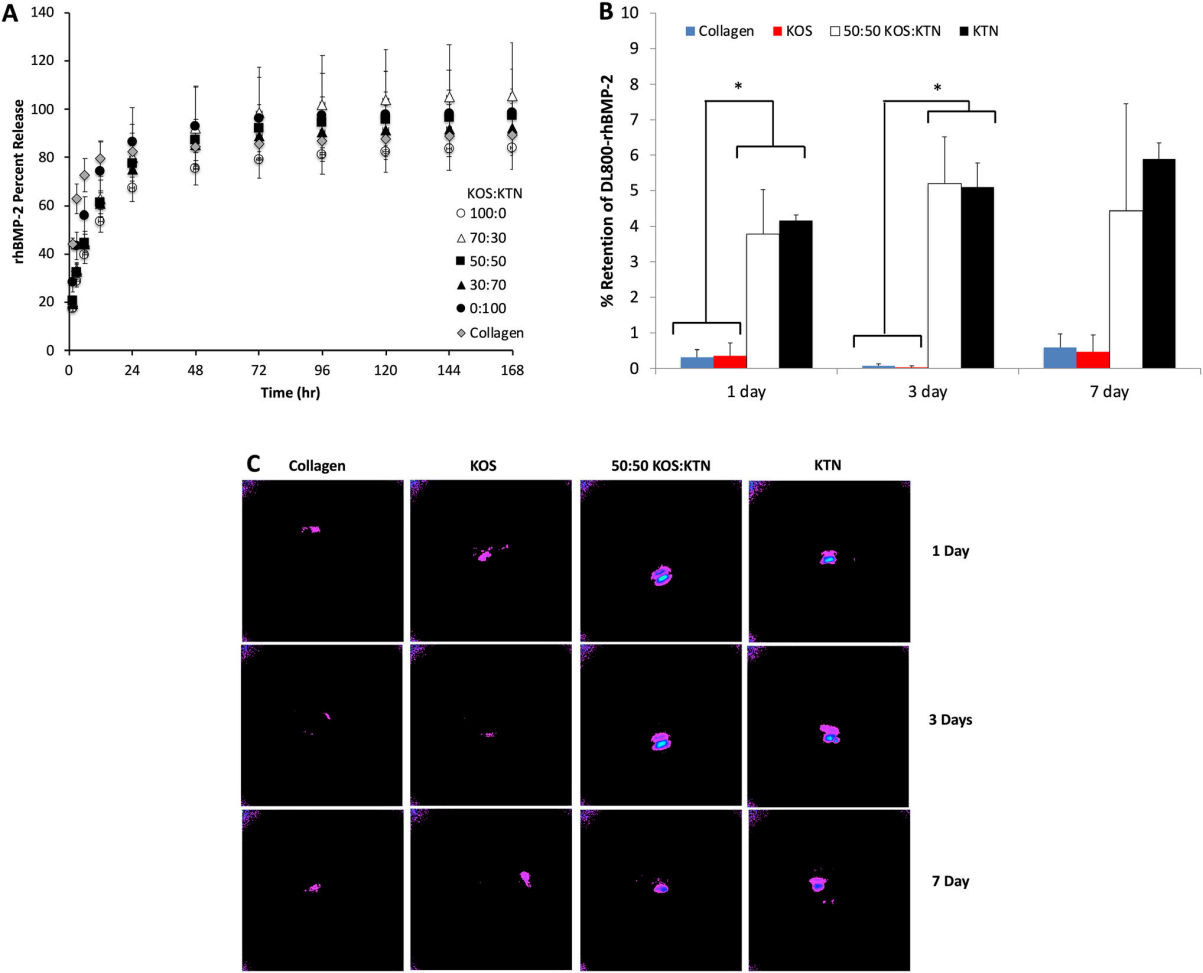
**A** In vitro release of AF488-rhBMP-2 from keratin hydrogels showing similar release for each of the formulations. **B** The average percent retention of DL800-rhBMP-2 at 1, 3, and 7 days. **C** Images of DL800-rhBMP-2 retention at femur defect site for three keratin formulations and for collagen at 1, 3, or 7 days post-implant. N = 4 for Day 1 and Day 3, N = 5 for Day 7 and error bars represent standard error of the mean

To better understand how the various formulations were impacting rhBMP-2 delivery and bone regeneration, we conducted *in vitro* and *in vivo* studies to investigate release of rhBMP-2 (*in vitro*) and retention of rhBMP-2 at the defect site (*in vivo*) with keratin collagen carriers. For the *in vitro* study, we labeled rhBMP-2 with AF488 rhBMP-2. The *in vitro* release profiles with AF488 rhBMP-2 (Fig. 5A) followed a similar trend to those found previously when using ELISA for rhBMP-2 detection [15]. Our interpretation of this *in vitro* result is that the fluorescent label does not significantly impact rhBMP-2 release from keratin materials.

For the *in vivo* studies, we adopted the use of DyLight800 as the fluorophore rather than AF488. DyLight800 fluoresces in the near-IR range, which we reasoned should reduce autofluorescence. For the *in vivo* study, we only investigated collagen, KOS, KTN, and the 50:50 KOS:KTN. We selected 50:50 KOS:KTN as it appeared to be the most promising keratin mixture formulation (see Figs. 1, 2,3 and 4). The use of fluorescence imaging and quantification used to generate the standard curve for determination of rhBMP-2 retention is outlined in Supplemental Fig. S1. As shown in Fig. 5 as quantified data (Fig. 5 B) or images (Fig. 5 C), all of the rhBMP-2 carriers (collagen, KOS, 50:50 KOS:KTN, and KTN) lost a large amount of rhBMP-2 *in vivo* within the first 24 h. However, the 50:50 KOS:KTN and the KTN carrier had significantly increased retention of rhBMP-2 at the 1-day and 3-day time points compared to KOS and collagen carriers. The retention of rhBMP-2 for the 50:50 KOS:KTN and the KTN carriers did not rise to the level of statistical significance at the 7-day time point due to greater variability in the retention. However, the results demonstrate that the rhBMP-2 retention is longer for the 50:50 KOS:KTN and KTN carriers.

**Fig. 6:**
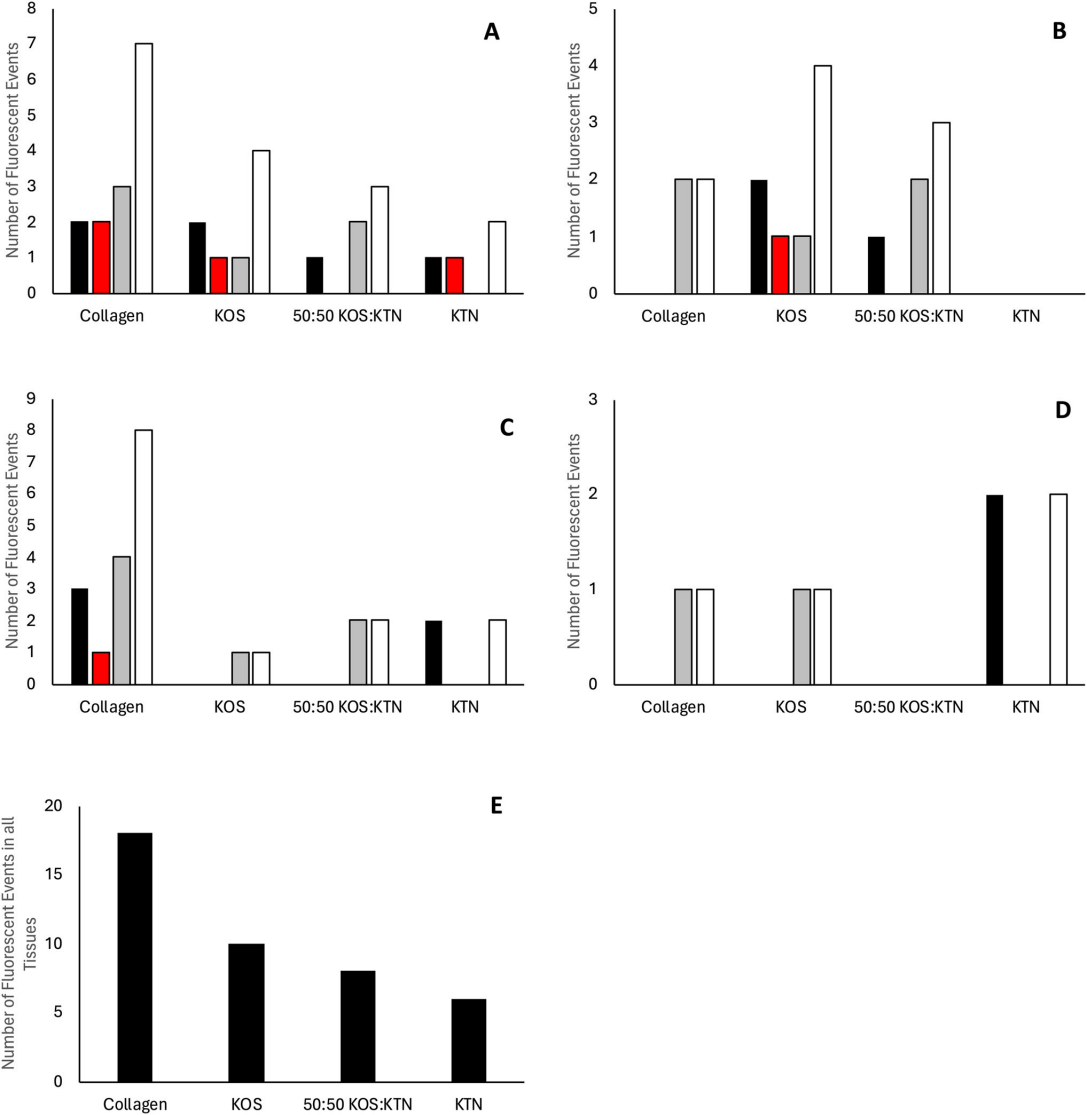
Summary of DL800-rhBMP-2 biodistribution as indicated by the number of fluorescent events in **A** spleen, **B** liver, **C** kidneys, **D** lungs at Day 1  , Day 3  , Day 7  , of the total () of all three days. N = 4 for Day 1 and Day 3, N = 5 for Day 7 and therefore N = 13 for the Total for both spleen and liver. For kidneys and lungs, both the left and right organs were observed, so N = 8 for Day 1 and Day 3 and N = 10 for Day 7.** E** ndicates the total of all fluorescent events for all tissues. N = 16 (4 for spleen, 4 for liver, 8 for kidney, 8 for lung) at Day 1 and Day 3 and N = 20 (5 for spleen, 5 for liver, 10 for kidney, 10 for lung) at Day 7

We also harvested other key organs (spleen, liver, kidneys, lungs, heart, and brain) from rats that had DL800-rhBMP-2 implant implanted at the femur defect site with collagen or keratin carriers. As summarized in Fig. 6A-D, we found detectable fluorescence in the spleen, liver, kidneys, and lungs for some carriers at each time point (see Supplemental Figs. S2, S3, S4, S5, S6 and S7 for images). We did not find fluorescence in the heart or brain tissues of any animals. We combined the data from what we refer to as “detectable events” for fluorescence and summarize this result in Fig. 6E. Figure 6E shows more detectable events in the spleen and liver with the collagen and KOS carriers compared to the 50:50 KOS:KTN and the KTN carriers. Moreover, there were more than double and triple the detectable events in all tissues with the collagen carrier compared to the 50:50 KOS:KTN and KTN carriers, respectively. There were also more total events with the KOS carrier than with the 50:50 KOS:KTN and KTN carriers. The number of detectable events in all tissues and in the spleen and liver are inversely correlated with retention at the defect site with a lower likelihood of detection at distal organ sites if more rhBMP-2 is retained at the defect site.

## Discussion

There are numerous strategies being investigated to achieve safer and more effective bone repair for nonunions including the use of biomimetic BMP-2 peptides [21], immobilization strategies for delivery of growth factors [22, 23], gene therapy [24, 25], and cell delivery [26, 27]. Most of these strategies could benefit from the approach described in this manuscript with keratin biomaterials. For example, keratins can be used as shear-thinning gels, which could allow for them to be injected in a minimally-invasive fashion. Because they are shear-thinning, keratins can also be 3D printed [28] or fabricated by other means into desired shapes to match, for example, patient-specific defects. Likewise, keratins have been used as carriers for cells [29] and numerous controlled release strategies [30, 31]. We have not described the characterization of the keratins as part of this report. However, keratins extracted from a similar source and by similar methods have previously been characterized by protein-induced X-ray emission for elemental analysis, amino acid analysis, thiol content, SEM for porosity, rheological properties, and mechanical properties [14, 32–34]. Keratin materials have received U.S. FDA approval for radiation dermatitis via the 510 k mechanism and have been used in human trials [35], suggesting a viable path to the clinic for bone applications. However, further analysis of the safety profiles of keratins in bone tissues would be beneficial.

Although we have previously described the use of keratin hydrogels, in this study we elected to freeze-dry keratin hydrogels that had been loaded with rhBMP-2. These scaffolds could be easily handled and implanted in the critically-sized femur defect, but alternative forms of the carriers can be used in the future. We investigated multiple outcomes measures for efficacy and safety, with some carriers performing better than others depending on the outcome assessed. Notably, at the 8-week time point, bridging of the defect site was not observed in all animals treated with KOS, KTN or 30:70 KOS:KTN formulations. However, BMD was comparable to or better than collagen for the keratin carriers and the 50:50 KOS:KTN carrier had greater bone volume than collagen. Likewise, max loads for KOS and 50:50 KOS:KTN were nearly equal to collagen while KOS had a higher elastic modulus than the collagen carrier. Differences were not statistically significant for most outcomes, suggesting that the keratin carriers were equivalent to collagen carriers by these metrics.

The results of the biodistribution study are particularly important in terms of both safety and efficacy. We have found little discussion in the literature about rhBMP-2 biodistribution in other locations following implantation in bone defects or impacts of such distribution to other tissues on safety profiles. It is particularly noteworthy that the experimental groups with the least retention at the femur defect implantation site (collagen and KOS carriers) had the most detectable fluorescence events in the distal tissues whereas samples with higher levels of retention (50:50 KOS:KTN and KTN carriers) had the least detectable signal. This is in spite of the fact that all of these formulations showed large amounts (95 – 99%) of rhBMP-2 loss by 1 day after implantation. Thus, retention of even relatively small amounts of rhBMP-2 by the KTN and 50:50 KOS:KTN formulations could improve safety profiles. BMP-2 has known roles in development [36], on tumorigenesis, [37] and in various body tissues [38–40]. We did not observe any gross changes in the tissues and others have reported minimal toxicity from systemic rhBMP-2 [41]. However, further characterization of the biological significance of rhBMP-2 in the distal organs would be beneficial in future studies.

Our use of a near-IR fluorophore coupled to rhBMP-2 should reduce the likelihood of the observed signals being associated with autofluorescence of the tissues [42]. Thus, the use of formulations with KTN may reduce the likelihood of side effects of rhBMP-2 reaching distal organs such as liver, spleen or kidney. In addition, increased retention of rhBMP-2 at the defect site would be expected to promote enhanced bone regeneration.

In prior work, we have characterized the rates of degradation of the KOS:KTN formulations that were used in this manuscript [14]. The degradation profiles correlated closely with the level of disulfide crosslinking where KOS-only and formulations with higher levels of KOS degraded more rapidly due to lower levels of disulfide crosslinking whereas KTN-only and formulations with higher levels of KTN degraded more slowly due to the presence of disulfide crosslinks [14]. In the present work in the femur defect model, we elected to use the same KOS:KTN ratios as in previous studies so that we could have some understanding of how the degradation profiles with these formulations might be impacting bone regeneration. However, there is no reason that other ratios of KOS:KTN could not be used in the future to further fine-tune degradation rates. We have also previously characterized how the different keratin formulations impact rhBMP-2 bioactivity with an *in vitro* alkaline phosphatase activity in MC3T3-E1 cells and an *in vivo* mouse heterotopic bone model [15]. This prior work showed that formulations with more KTN had higher levels of rhBMP-2 bioactivity than formulations with more KOS. These prior studies thus suggest that there is a balance between degradation rate and bioactivity of the rhBMP-2 delivered from keratin carriers.

**Fig. 7:**
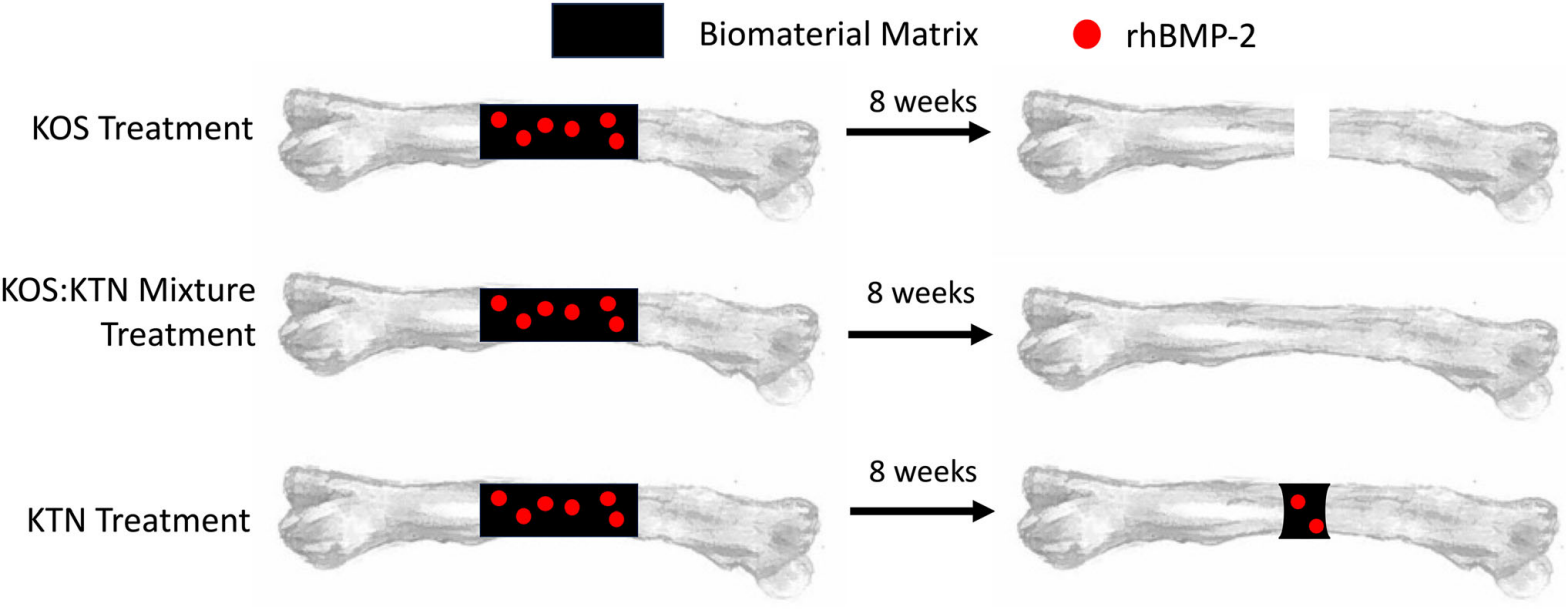
Possible mechanism of action for keratin (or collagen) carriers of rhBMP-2, showing impacts of biomaterial degradation and rhBMP-2 retention. Initially (top) all formulations have equal amounts of the material carrier (collagen, KOS, KOS:KTN mixtures, or KTN) and rhBMP-2. After time (bottom panel), KOS has rapid loss of the biomaterial at the implant site (white color) with time due to the lack of disulfide crosslinking. This loss of the material carrier leads to loss of rhBMP-2 from the implant that promotes rapid bone formation but with both local (ectopic growth at implant site) and systemic (rhBMP-2 in distal organ) effects. Some aspects of this (rapid degradation and rhBMP-2 in distal organs) is also true for collagen carriers. After time, KTN (bottom, right) has greater retention of rhBMP-2 at the implant site. However, the slow degradation of KTN may impede bridging because KTN remains at the implant site. The KTN:KOS mixtures (bottom, center) may provide some benefits of both systems. The degradation of KOS may allow cellular infiltration and subsequent bone bridging with rhBMP-2 release locally. However, the presence of KTN may aid in retention of more rhBMP-2, lessening local and especially systemic effects

In Fig. 7, we have attempted to illustrate how we believe that mixtures of KOS and KTN may be more advantageous than the use of these forms of keratin used alone based on the degradation and rhBMP-2 bioactivities noted above. Specifically, KTN may enhance retention and bioactivity, but if used as the only component of the formulation may impede full bone regeneration and bridging due to its slow rate of degradation. Indeed, we observed residual KTN at the defect site grossly, during mechanical testing (Supplemental Fig. S8 part I; white arrow), and histologically (Supplemental Fig. S9, arrow), though we did not observe obvious signs of inflammatory response around the residual keratin material (Supplemental Fig. S10). Conversely, the KOS may lead to high levels of osteoinduction due to the rapid release of rhBMP-2 from the carrier, but when used alone may degrade too quickly giving lower BMD values and higher levels of rhBMP-2 release and distribution to distal organs. It is interesting that collagen supported bridging in all animals while KOS did not, but previously known issues of porous bone with collagen carriers of rhBMP-2 were observed even though the defects were bridged. In addition, the collagen carriers had the most rhBMP-2 in distal organs of those scaffolds investigated in this study.

We suggest that the use of KOS:KTN mixtures may offer the benefits of KOS and KTN; namely, suitable rates of material degradation and rhBMP-2 retention. Because KTN has been shown to enhance the bioactivity of rhBMP-2 *in vitro* compared to collagen carriers [15], it may be possible to use reduced doses of rhBMP-2 in the future with KTN-based materials. We cannot rule out the possibility that the freeze-drying process could decrease rhBMP-2 activity and we did not use cryopreservation methods that have been reported to enhance rhBMP-2 bioactivity [43]. Others have recently shown that the methods of freeze-drying can be used to impact pore structure of keratin scaffolds that led to improved healing in a cranial rat defect model [44]. Thus, future improvements in the keratin carriers could be achieved through relatively minor adjustments to the keratin formulation and processing methods. Future studies that investigate sex differences on mechanisms and outcomes of bone healing in response to rhBMP-2 from keratin biomaterials would also be beneficial. However, we are not aware of studies on sex-based differences in response to rhBMP-2 in humans, and we suggest that the safety and efficacy mechanisms that were the focus of the current manuscript are likely to benefit both males and females.

Overall, the results of our studies show that the flexibility of keratin carriers in terms of tunable rates of degradation and impacts on rhBMP-2 bioactivity and retention at the defect site are an advantageous platform for rhBMP-2 delivery. The results demonstrate the importance of both material degradation rates and rhBMP-2 retention and release on bone formation and point to more general approaches, beyond keratin carriers, that could improve the future of off-the-shelf approaches to repairs of delayed and nonunion fractures in long bones.

Keratin carriers of rhBMP-2 can promote bone regeneration in critically-sized defects in long bones. The keratin carriers performed as well as or better than collagen carriers by several metrics of efficacy. Mixtures of KOS and KTN may provide advantages associated with each material alone but mitigate problems with the individual keratin formulations. Importantly, we have demonstrated that certain keratin carriers (those with KTN) may enhance safety profiles through enhanced retention of the rhBMP-2 at the defect site.

## Supplementary Information

Below is the link to the electronic supplementary material.

Supplementary file1 (TIFF 386073 kb) Approach to creating standard curve for DL800-rhBMP-2 at femur defect site on Bruker Multispectral Imaging System showing the X-ray, optical image, fluorescence intensity images for 100 μg/mL (14.2 μg) rhBMP-2 standard (top row) and one image of 3 total standards for 100, 50, 25, 12.5, 6.25, 3.125, 1.5625 and 0 μ/mL (14.2, 7.25, 3.625, 1.813, 0.906, 0.453, 0.227, and 0 μg) DL800-rhBMP-2 (bottom row)

Supplementary file2 (TIFF 154130 kb) Bruker Multispectral Imaging System images of explanted spleen at 1, 3 and 7 days showing detectable DL800-rhBMP-2 in some samples (pink)

Supplementary file3 (TIFF 128996 kb) Bruker Multispectral Imaging System images of explanted liver at 1, 3 and 7 days showing detectable DL800-rhBMP-2 in some samples (pink)

Supplementary file4 (TIFF 203968 kb) Bruker Multispectral Imaging System images of explanted lung at 1, 3 and 7 days showing detectable DL800-rhBMP-2 in some samples (pink)

Supplementary file5 (TIFF 195415 kb) Bruker Multispectral Imaging System images of explanted kidney at 1, 3 and 7 days showing detectable DL800-rhBMP-2 in some samples (pink)

Supplementary file6 (TIFF 143525 kb) Bruker Multispectral Imaging System images of explanted heart at 1, 3 and 7 days showing no detectable DL800-rhBMP-2 in any of the samples

Supplementary file7 (TIFF 121926 kb) Bruker Multispectral Imaging System images of explanted brain at 1, 3 and 7 days showing no detectable DL800-rhBMP-2 in any of the samples

Supplementary file8 (TIFF 159147 kb) Explanted bone tissue prior to mechanical testing. (A) No treatment/empty, (B) KOS-only/no rhBMP-2, (C) KTN-only/no rhBMP-2, (D) collagen + rhBMP-2, (E) KOS + rhBMP-2, (F) 70:30 KOS:KTN + rhBMP-2, (G) 50:50 KOS:KTN + rhBMP-2, (H) 30:70 KOS:KTN + rhBMP-2, (I) KTN + rhBMP-2. Scale bar indicates 1 cm. Arrow in (I) indicates residual KTN material observed at implant site

Supplementary file9 (TIFF 325914 kb) Masson’s trichrome staining of left femurs for collagen + rhBMP-2, KOS + rhBMP-2, 70:30 KOS:KTN + rhBMP-2, 50:50 KOS:KTN + rhBMP-2, 30:70 KOS:KTN + rhBMP-2, KTN + rhBMP-2 and KOS + rhBMP-2. Scale bar indicated 1 mm. Arrow indicates residual KTN material at implant site

Supplementary file10 (TIFF 83160 kb) H&E staining of left femurs for collagen + rhBMP-2, KOS + rhBMP-2, 70:30 KOS:KTN + rhBMP-2, 50:50 KOS:KTN + rhBMP-2, 30:70 KOS:KTN + rhBMP-2, KTN + rhBMP-2 and KOS + rhBMP-2. Scale bar indicated 1 mm

## Data Availability

The data that supports the findings in this study are available from the corresponding authors upon reasonable request.
